# Trigeminal neuralgia post-styloidectomy in Eagle syndrome: a case report

**DOI:** 10.1186/1752-1947-6-333

**Published:** 2012-10-02

**Authors:** John William Blackett, Daniel J Ferraro, John J Stephens, Joshua L Dowling, Jerry J Jaboin

**Affiliations:** 1 Department of Radiation Oncology, Mallinckrodt Institute of Radiology and Siteman Cancer Center, Washington University Medical School, 4511 Forest Park Avenue, Saint Louis, MO 63108, USA; 2 Division of Neuroradiology, Mallinckrodt Institute of Radiology, Washington University Medical Center, St. Louis, MO 63110, USA; 3 Department of Neurological Surgery, Washington University Medical School, 660S. Euclid Avenue, Campus Box 8057, Saint Louis, MO 63110, USA

## Abstract

**Introduction:**

Eagle syndrome is a condition characterized by an elongated (>3cm) styloid process with associated symptoms of recurrent facial or throat pain. In this report we present a case of Eagle syndrome exhibiting the typical findings of glossopharyngeal nerve involvement, as well as unusual involvement of the trigeminal nerve. Notably, this patient developed a classical trigeminal neuralgia post-styloidectomy.

**Case presentation:**

A 68-year-old Caucasian woman presented with a 25-year history of dull pain along the right side of her throat, lateral neck, and jaw. Her symptoms were poorly controlled with medication until 15 years ago when she was diagnosed with Eagle syndrome, and underwent a manual fracture of her styloid process. This provided symptomatic relief until 5 years ago when the pain recurred and progressed. She underwent a styloidectomy via a lateral neck approach, which resolved the pain once again. However, 6 months ago a new onset of triggerable, electric shock-like facial pain began within the right V1 and V2 distributions.

**Conclusions:**

Eagle syndrome is distressing to patients and often difficult to diagnose due to its wide variability in symptoms. It is easily confused with dental pain or temporomandibular joint disorder, leading to missed diagnoses and unnecessary procedures. Pain along the jaw and temple is an unusual but possible consequence of Eagle syndrome. An elongated styloid process should be considered a possible etiology of dull facial pain in the trigeminal distributions, in particular V3.

## Introduction

Eagle syndrome is characterized by recurrent unilateral pain in the oropharynx that may radiate to the ear, cheek, jaw, eye, or neck. It is usually described as dull and constant [1,2]. Other common symptoms include dysphagia, globus pharyngis, odynophagia or pain with rotation of the head [3-5]. It is uncommonly reported, but probably a more common source of facial and throat pain than realized [5,6].

Eagle syndrome is caused by an elongated styloid process that compresses nearby structures such as nerves and blood vessels. Typical styloid processes are between 2.5 and 3.0cm in length. Lengths greater than 3cm are considered elongated [6,7]. Bilateral elongation is common, although symptoms are typically unilateral [5,7]. A review of 52 cases of Eagle syndrome found that 50% of patients had bilateral elongation, but only half of those cases had bilateral symptoms [4]. In most cases, resection of the styloid process resolves the pain [1,2].

In this case we review a patient with elongated styloid processes exhibiting classical symptoms of Eagle syndrome along with a more unusual pain in the V3 distribution. Her pain was resolved by a styloidectomy for several years but, more recently, she has experienced symptoms of trigeminal neuralgia in the V1 and V2 regions. In our report we review the anatomy of the stylohyoid complex as well as the pathophysiology of Eagle syndrome.

## Case presentation

Our patient is a 68-year-old Caucasian woman with a past medical history significant for a tonsillectomy 50 years ago. She developed a constant, dull and severe right-sided pain in her throat, neck, teeth and jaw along with dysphagia beginning approximately 25 years ago.

At the outset she attributed her symptoms to dental origins so she underwent tooth extractions on the right side without relief of pain. She was initiated on multiple pain medications, but her pain was improved though poorly controlled with carbamazepine, baclofen, and high doses of opiates. Ten years following the initial onset of her pain she was evaluated by an oromaxillofacial surgeon who noted that her symptoms were consistent with Eagle syndrome. He performed a manual fracture of her styloid process, which provided nearly instantaneous pain relief for 10 years, at which time she experienced a recurrence of her symptoms.

**Figure 1:**
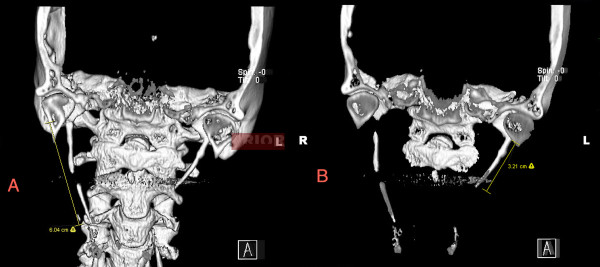
**Three-dimensional reconstruction of pre-styloidectomy maxillofacial computed tomography scan showing elongated styloid processes.** (**A**) The right styloid process is extremely elongated, at approximately 6cm. The tip of the right styloid process has been separated from the main process as a result of the 1997 manual fracture procedure. The remaining superior portion of the right styloid process is similar in length to the left styloid process. (**B**) The left styloid process is moderately elongated at 3.2cm.

At that time she underwent a computed tomography scan of the maxillofacial structures which revealed bilateral elongated styloid processes, particularly on the right, and she was officially diagnosed with Eagle syndrome. The extremely elongated right styloid process (approximately 6cm) and moderately elongated left styloid process (3.2cm) can be seen in Figure 1. A styloidectomy using an external approach significantly relieved her pain, and she was able to discontinue her pain medication regimen.

Two years after her styloidectomy the patient had an outbreak of shingles in her right ophthalmic division; the shingles healed without incident and left no residual pain or alteration of sensation. Five years post-styloidectomy she developed right-sided facial pain extending from her upper lip to the middle of her forehead. She described her pain as electric shock-like and transient. It was triggered by light touch and activities such as brushing teeth, talking, and blowing her nose.

She restarted 800 to 1600mg carbamazepine and up to 10mg baclofen daily, however, she took these medications irregularly due to side effects that interfere with work and activities of daily living. Due to the medications she typically slept for 12 hours each day after work, and the full-dose baclofen affected her balance and gait. Although the side effects were significant, the medications reduced the frequency and severity of her episodes.

**Figure 2:**
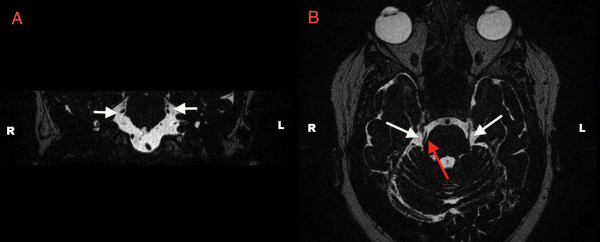
**Recent magnetic resonance image showing the trigeminal nerve roots.** In Panel **A** (coronal view) and **B** (axial view), the trigeminal nerves enter the brainstem at a region referred to as the trigeminal nerve root entry zone (white arrows delineate the trigeminal nerves). In Panel **A** (coronal view), the right trigeminal nerve and right superior cerebellar artery are overlapped on the right, and can be visualized as two separate structures on the left. In Panel **B** (axial view), the right superior cerebellar artery can be seen adjacent to the right trigeminal nerve as it enters the brainstem.

Following the development of her right-sided facial pain, she underwent magnetic resonance imaging of the brain and brainstem, which revealed scattered T2 hyperintensities in the deep white matter, a non-specific finding. No cranial nerve lesions were apparent. A loop of superior cerebellar artery was in contact with the right trigeminal nerve root, which can be seen in Figure 2.

Because of inadequate pain relief and medication side effects, the patient was referred for possible surgical management. The various surgical treatment options were considered and discussed with the patient, including microvascular decompression, percutaneous rhizolysis, and Gamma Knife® radiosurgery. She elected to pursue Gamma Knife® radiosurgery with the intent to taper oral pain medication as her pain subsides over time. No deficits were detected in her cranial nerves and pain was not elicited with touch or percussion. Otherwise, her physical examination was negative.

## Discussion

The temporal styloid process is a slender bone lying just below the ear, anterior to the mastoid process and between the internal and external carotid arteries, with the internal jugular vein and the glossopharyngeal, vagus, hypoglossal, and accessory nerves lying medial. Two ligaments (the stylohyoid and stylomandibular) and three muscles (the stylopharyngeus, stylohyoid, and styloglossus) attach to the styloid process [5].

W. W. Eagle first described Eagle syndrome in 1937 among patients who had undergone tonsillectomy [1-3]. Trauma or surgery can lead to ossification or elongation of the stylohyoid complex, which can then impinge upon cranial nerves V, VII, IX, X, and XI, all of which pass near the styloid process [7]. Genetic polymorphism and early onset menopause have also been suggested as causes of stylohyoid ossification. Eagle syndrome is most commonly diagnosed in women >30 years old [2]. Our patient underwent a tonsillectomy in 1962, about 25 years before her pain began. This is an unusually long interval between the procedure and the onset of symptoms. Eagle described the symptoms occurring immediately after tonsillectomy due to stretching or fibrosis in the sensory nerve endings of the fifth, seventh, ninth, and tenth cranial nerves [8].

Eagle syndrome can be divided into two categories [7-9]. In the first, the elongated styloid process compresses cranial nerves, often the glossopharyngeal nerve [1], resulting in throat and neck pain. In the second category, carotid artery syndrome, the styloid process compresses the internal carotid artery, which may cause transient ischemic attacks [9] or compression of the sympathetic nerves running along the artery, leading to a variety of symptoms [3,7]. The pain in Eagle syndrome often resembles glossopharyngeal neuralgia, but is typically more dull and constant [1]. Although there have been reports describing it as a sharp intermittent pain along the path of the glossopharyngeal nerve [7]. Several mechanisms have been suggested to explain the pain caused by an elongated styloid process. These include compression of nearby nerves, such as the glossopharyngeal, chorda tympani, and lower branch of the trigeminal; fracture of the ossified stylohyoid ligament; compression of the internal carotid artery and sympathetic nerves; degenerative and inflammatory changes in the stylohyoid insertion; irritation of the pharyngeal mucosa by the elongated styloid process; and stretching and fibrosis of cranial nerves V, VII, IX, and X post-tonsillectomy [2,8].

Typically Eagle syndrome pain is characterized by pharyngeal pain in the tonsillar fossa, sometimes radiating up to the hyoid bone [6]. It has been described as a dull nagging pain in the throat, sometimes radiating to the ear or mastoid [5]. In a review of 52 cases of Eagle syndrome, 22 reported pain in the neck, and 19 reported pain in the throat. Only seven patients had pain upon swallowing, and just one had pain in the jaw [4]. Eagle syndrome may be treated conservatively with medication or through surgical shortening of the styloid process. Surgical resection through either an intraoral or external approach is considered the most effective treatment [2].

This patient is particularly interesting due to her complex history of Eagle syndrome and trigeminal neuralgia. She originally presented with typical symptoms of Eagle syndrome: dull aching pain in the throat and neck that was exacerbated by swallowing. This suggests impingement of the glossopharyngeal nerve by an elongated styloid process. However, she also exhibited symptoms that suggest mandibular nerve involvement, such as pain along the jaw line and temple.

Her right styloid process was also exceptionally long at nearly 6cm. An elongated or ossified styloid process typically impinges upon the glossopharyngeal nerve causing the characteristic oropharynx pain. However, it can also affect the lower branch of the trigeminal nerve, leading to pain in the V3 distribution along the jaw and temple as in our patient [2,4,5]. The presence of pain along the jaw line pre-styloidectomy suggests that the patient’s styloid process was impinging upon the mandibular nerve in addition to the glossopharyngeal nerve. This is somewhat unusual because the mandibular nerve is not often affected in Eagle syndrome. The contact between our patient’s superior cerebellar artery and trigeminal nerve root may have sensitized the V3 branch of her trigeminal nerve to compression or stretching by the elongated styloid process and surrounding scar tissue, resulting in the unusual jaw pain. If so, resection of the styloid process would have relieved compression of the glossopharyngeal and mandibular nerves resulting in pain relief. However, it would not have prevented progression of the superior cerebellar artery’s compression of the trigeminal root, which was the cause of her current trigeminal neuralgia (compression of the trigeminal nerve by the superior cerebellar artery has been described as a common cause of trigeminal neuralgia [10]).

Our patient’s experience of dull constant jaw pain followed by typical trigeminal neuralgia is similar to descriptions of pre-trigeminal neuralgia. Patients with this prodromal pain describe a dull constant ache in the upper or lower jaw, which is followed by the classic paroxysmal pain of trigeminal neuralgia [11]. The initial pain is often mistaken for a toothache or temporomandibular joint disorder. Typical trigeminal neuralgia develops days to years after the onset of the initial pain sometimes with an asymptomatic interval. Often there is temporary relief after an intervention to relieve the jaw pain is carried out. A case series on pre-trigeminal neuralgia describes a patient whose pain was relieved by vagal body excision for 1 month, and another patient whose pain was relieved by an intraoral orthosis for 7 to 8 months. In both cases this pain-free period was followed by typical trigeminal neuralgia [11]. In another series of 38 patients experiencing pre-trigeminal neuralgia, six underwent surgical excision of tooth apices to relieve their jaw pain. Five of these patients experienced temporary relief for periods of 2 months to a year, one of whom experienced a recurrence of pain of the same character and four experienced classical symptoms of trigeminal neuralgia [12].

This suggests that trigeminal neuralgia often exists in a prodromal state characterized by dull constant jaw pain, particularly if there is a distal structural defect to exacerbate the pain. The underlying abnormality that eventually causes classical trigeminal neuralgia may be present in an early form. This early form might not be capable of causing facial pain on its own, but might sensitize distal trigeminal nerve branches to local compression or stretching. It might sensitize the alveolar nerves to pain in the jaw from dental problems or in our case from an elongated styloid process and scar tissue.

The patient had an outbreak of shingles between her surgery and the onset of trigeminal neuralgia, but showed no signs of postherpetic neuralgia. Postherpetic trigeminal neuralgia is characterized by pain persisting after an outbreak of herpes zoster. Although thoracic dermatomes are most commonly affected, the trigeminal nerve is the next most common location. The pain usually accompanies the vesicular eruption but lasts only for this period. Between 10% and 20% of patients will suffer from postherpetic pain. The incidence of postherpetic pain increases with age [13]. Definitions of postherpetic neuralgia are arbitrary and include development from 1 to 6 months after the rash [14]. Rare cases of delayed onset postherpetic neuralgia have been reported occurring months to years after the initial rash, but in those cases the pain was triggered by an event such as surgery [15].

## Conclusion

Eagle syndrome is a complex condition caused by an elongated styloid process that is associated with a wide variety of symptoms. Although most commonly associated with throat and neck pain that is worsened by head rotation, swallowing, or chewing, it may also be associated with pain in a trigeminal V3 distribution. Although the glossopharyngeal nerve is most commonly implicated in Eagle syndrome, involvement of the mandibular nerve is possible. Eagle syndrome should be considered a possible etiology of dull pain along the jaw line or temple. Pain in this distribution is an uncommon but possible symptom of Eagle syndrome that is easily confused with other sources of facial discomfort, such as temporomandibular joint disorder or dental pain.

## Consent

Written informed consent was obtained from the patient for publication of this case report and accompanying images. A copy of the written consent is available for review by the Editor-in-Chief of this journal.

## Competing interests

The authors declare that they have no competing interests.

## Authors’ contributions

The patient was under the care of JJ and JLD. Imaging was reviewed by JJS. JJ contacted the patient for consent. JWB, DF, JLD and JJ drafted the manuscript. All authors reviewed and approved the manuscript prior to publication.
